# Live imaging *Arabidopsis thaliana* embryos under different hydration conditions

**DOI:** 10.1016/j.xpro.2021.101025

**Published:** 2021-12-13

**Authors:** Yanniv Dorone, Steven Boeynaems, Seung Y. Rhee

**Affiliations:** 1 Department of Plant Biology, Carnegie Institution for Science, Stanford, CA 94305, USA; 2 Department of Biology, Stanford University, Stanford, CA 94305, USA; 3 Department of Genetics, Stanford University School of Medicine, Stanford, CA 94305, USA

**Keywords:** Cell Biology, Developmental biology, Microscopy, Model Organisms, Plant sciences, Molecular Biology

## Abstract

Despite the ecological and agronomical importance of seed germination, how seeds integrate environmental signals to trigger germination remains enigmatic. Recently we reported that a protein called FLOE1 is involved in sensing and responding to water availability during germination. Here, we present a live-imaging protocol to assess the subcellular localization of a protein of interest during imbibition of desiccated *Arabidopsis thaliana* seeds with the goal of understanding protein dynamics during the early stages of water uptake.

For complete details on the use and execution of this profile, please refer to Dorone et al. (2021).

## Highlights

• Steps for *Arabidopsis thaliana* embryo dissection
• Protocol for confocal imaging of embryos
• Analysis of protein localization under different levels of water potential

## Graphical abstract

**Figure:**
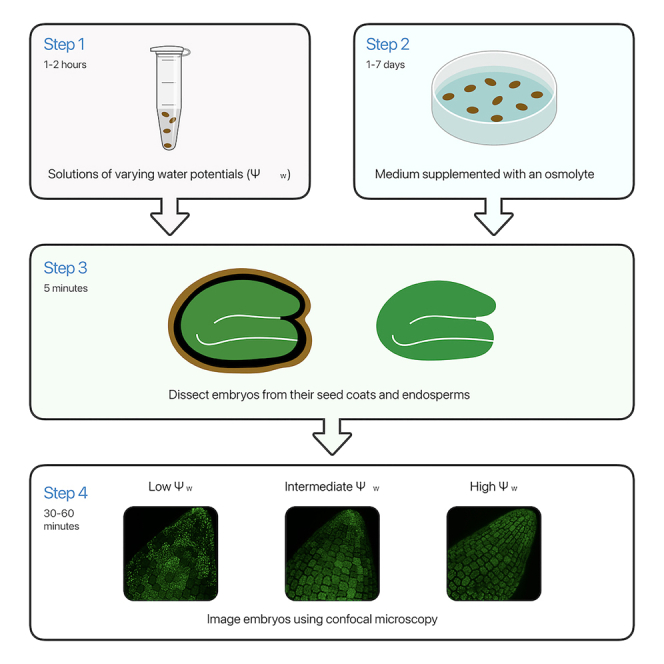

## Before you begin

The process of seed germination spans the initial uptake of water and ends with the protrusion of the radicle (Steinbrecher and Leubner-Metzger, 2018). Water uptake can be broken into three main phases based on the water content of the seed (Gallardo et al., 2001). Phase I is characterized by rapid water uptake, and is quickly followed by a plateau phase (Phase II) during which water content stays stable. In Phase III, water uptake increases again and enables radicle emergence through cellular growth. Germination *sensu stricto* refers to the first two phases, during which seeds maintain their desiccation tolerance (Gallardo et al., 2001). Up until Phase III, seeds may undergo numerous cycles of hydration-dehydration while maintaining their germination potential (Bai et al., 2012). However, the molecular details behind whether a seed will enter Phase III remain unresolved.

As illustrated by the example of the germination regulator FLOE1 which changes its biophysical state and subcellular localization in response to water (Dorone et al., 2021), a key element of this puzzle is understanding protein dynamics such as liquid-liquid phase separation and protein localization *in vivo* in response to water potential. Assessing protein dynamics in embryos *in vivo* is complicated by multiple confounding factors. First, embryos are protected by a seed coat that serves as a physical barrier that slows down water entry (Debeaujon et al., 2007). Therefore, the water potential (Ψ_w_) of the environment surrounding the seed may not reflect the water potential perceived by the embryo at a given point. In this protocol, we describe two complementary approaches to assess protein localization in response to varying levels of water potential: one that entails removing the seed coat and directly exposing naked embryos, and another that captures the state of the embryo prior to removal of the seed coat. Secondly, embryo cells contain highly autofluorescent protein storage vacuoles, which may compromise interpretations of subcellular localization of fluorescent proteins that are not expressed at high levels. This drawback is compounded by the fact that the level of autofluorescence is affected by water potential. We indeed found in Dorone et al. (2021) that protein storage vacuoles appear more autofluorescent when water potential is low. Therefore, the fluorescence needs to be normalized to compare fluorescent protein localization under different levels of water potential. By using the example of embryos expressing FLOE1p::FLOE1-GFP , we describe such a normalization method.

### Obtaining *Arabidopsis* seeds

**Timing: 2–3 months**

Because environmental maternal effects can significantly impact seed characteristics such as weight, germination ability, and dormancy (Jaganathan, 2016; Wulff, 2017), it is important to grow plants in a consistent and controlled manner to ensure reproducibility of the results. This section is intended to specify best practices when growing mother plants to reduce environmental effects on the seed progeny. For more general protocols on growing *A. thaliana*, see (Rivero et al., 2014).

1. To ensure synchronous germination, stratify seeds from the fluorescently tagged lines and a wild-type control (e.g., Col-0) as follows:
  a. Submerge seeds in double-distilled water in 1.5 mL Eppendorf tubes. Properly submerged seeds should sink to the bottom. If they float, invert the tubes multiple times until all seeds sink to the bottom.
  b. Cover tubes with aluminum foil.
  c. Place tubes at 4°C for 3–5 days.
2. Sow seeds in individual pots of soil (e.g., PRO-MIX® HP Mycorrhizae) and cover with a plastic dome. We recommend growing at least six plants per genotype.
3. Transfer pots to a controlled environment, preferably a growth cabinet programmed to the desired growth settings (e.g., 16 h light/8 h dark at 22°C, 130 μmol m^−2^ s^−1^ Photosynthetic Photon Flux Density (PPFD), 50%–60% relative humidity). For optimal conditions, see the Arabidopsis Biological Resource Center (ABRC) recommendations at ftp://ftp.arabidopsis.org/ABRC/abrc_plant_growth.pdf.

***Note:*** Since seed characteristics can be dramatically altered by the local microenvironment during maturation on the mother plants (Gutterman, 2000), it is important to use a growth system (e.g., growth cabinet) to precisely control growth conditions. We recommend placing a data logger (e.g., HOBO temp/RH/light data logger) in the chamber, which can measure environmental parameters—including temperature, light and humidity—for the entire duration of the experiment.

4. To avoid any position effects within the growth chambers and to ensure population homogeneity (Measures et al., 1973), pots should be systematically randomized and rotated daily. The dome should be removed 5–7 days after germination.
5. When siliques begin to mature (indicated by their yellowing), relative humidity should be decreased to less than 50% as recommended by ABRC (ftp://ftp.arabidopsis.org/ABRC/abrc_plant_growth.pdf).

6. To ensure that seeds from the same time windows of fertilization are compared, harvest brown siliques in 3 batches:
  a. Batch 1 should be harvested two weeks after the first brown siliques appeared.
  b. Batch 2 should be harvested two weeks later.
  c. Batch 3 should be harvested after the plants are completely senesced.
    ***Note:*** We recommend harvesting seeds from each individual plant separately so that they can each serve as biological replicates.
  d. After air-drying seeds in open 1.5 mL Eppendorf tubes for a week at 20% relative humidity (RH) in a controlled cabinet, seeds should be transferred to cold storage (4°C) with RH ≤ 20%. When properly stored, seeds should last for years (Rivero et al., 2014).
    **CRITICAL:***Arabidopsis* seeds may quickly rehydrate if exposed to high humidity (Rivero et al., 2014), which will affect the results of this protocol. When taking seeds out of cold storage, tubes should be equilibrated at room temperature (20°C–23°C) before opening to prevent condensation from occurring around the seeds. When putting seeds back in storage, tubes should be left open in a 20% RH environment before closing to avoid trapping any moisture in. If a walk-in seed storage room (4°C and 20% RH) is available, it is preferable to work with aliquots taken directly inside the room to avoid subjecting the seed stocks to environmental fluctuations.

## Key resources table

**Table:** 

REAGENT or RESOURCE	SOURCE	IDENTIFIER
**Chemicals, peptides, and recombinant proteins**		

Glycerin	MilliporeSigma	Cat. # G2289
Sodium chloride	MilliporeSigma	Cat. # S3014

**Experimental models: Organisms/strains**		

*Arabidopsis thaliana*: Col-0	Arabidopsis Biological Resource Center (ABRC)	CS70000
*Arabidopsis thaliana*: FLOE1p::FLOE1-GFP;*floe1-1/floe1-1* (Col-0)	(Dorone et al., 2021)	N/A
*Arabidopsis thaliana*: 35S::FLOE1-GFP;*floe1-1/floe1-1* (Col-0)	(Dorone et al., 2021)	N/A

**Software and algorithms**		

Leica Application Suite X (LAS X)	Leica Microsystems	RRID: SCR_013673 (https://www.leica-microsystems.com/products/microscope-software/p/leica-las-x-ls/)
GraphPad Prism (v8.4.1)	GraphPad Software	RRID: SCR_002798 (https://www.graphpad.com/)

**Other**		

Nickel Plated Pin Holder	Fine Science Tools	Cat. # 26018-17
Tungsten Needles (rod diameter: 0.125 mm)	Fine Science Tools	Cat. # 10130-05
Leica TCS SP8 Confocal Microscope with HC PL APO CS2 63x/1.20 WATER and 63/1.30 GLYCERIN objectives.	Leica Microsystems	N/A

## Step-by-step method details

### Direct imbibition of embryos

**Timing: 1–2 h**

In this step, naked embryos are directly exposed to solutions of varying levels of water potential.

1. Prepare solutions of varying levels of water potential.
  a. Prepare a stock solution containing a high concentration of an osmolyte of your choice that will be used to modify the water potential. For example, in Dorone et al., (2021), we used NaCl, mannitol and sorbitol. In the case of NaCl, a solution of up to 6 M can be prepared by mixing 17.532 g NaCl (Sigma-Aldrich) in double-distilled water and adjusting the volume to 50 mL.
    ***Note:*** It is best to first prepare a high concentration of the osmolyte (without surpassing its maximum solubility) as well as a high volume to minimize variabilities between experiments. In the example above, we chose 6 M and 50 mL NaCl which will be used as a stock solution in the next step to generate 2 mL solutions of 0.2–1.8 M.
  b. Prepare dilutions of the stock solutions. In the case of NaCl, prepare solutions in increments of 200 mM from 0 M to 2 M. To mitigate pipetting inaccuracies, we recommend preparing first a 10 mL secondary stock of 2 M, and then preparing solutions of 2 mL using the following table:

**Table:** Dilutions of NaCl stock solution

Concentration	0.2 M	0.4 M	0.6 M	0.8 M	1 M	1.2 M	1.4 M	1.6 M	1.8 M
**2 M stock**	0.2 mL	0.4 mL	0.6 mL	0.8 mL	1 mL	1.2 mL	1.4 mL	1.6 mL	1.8 mL
**Water**	1.8 mL	1.6 mL	1.4 mL	1.2 mL	1 mL	0.8 mL	0.6 mL	0.4 mL	0.2 mL

    ***Note:*** We found in (Dorone et al., 2021) that exposure of embryos to 2 M NaCl induces a similar subcellular localization of FLOE1 as in the dry state, and therefore chose 2 M NaCl as the upper bound for the serial dilutions. However, depending on the protein of interest, the upper bound may be different and this parameter will need to be determined empirically.

**Figure 1:**
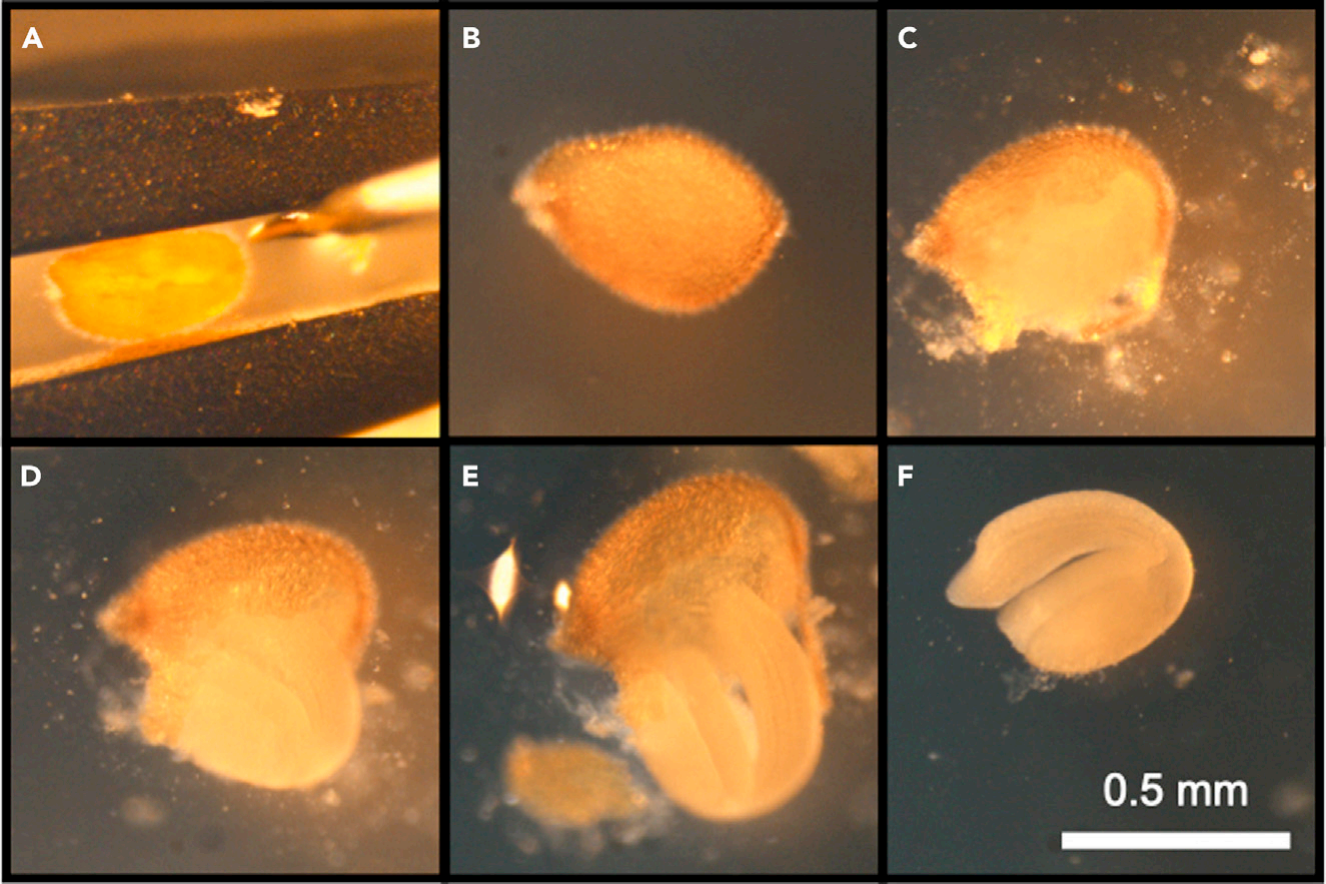
Dissection of an *Arabidopsis thaliana* seed (A) An *Arabidopsis* seed is held between tweezer tips while its seed coat and endosperm are being scraped off with a tungsten needle. (B–F) The same seed is imaged at different stages of dissection. A cavity is first created by superficially scraping the seed coat and endosperm off. Then, the embryo is gently pushed through it. Scale bar for (B–F) is shown in F.
  c. Using a tungsten needle held with a pin holder (see key resources table), superficially scrape the seed coat and endosperm without damaging the embryo to create a large enough cavity for the embryo to pass through (Figures 1B and 1C).
  d. Gently push the embryo out of the cavity to remove the seed coat and endosperm (Figures 1D–1F).
    ***Note:*** This step can be particularly challenging when dissecting seeds in non-aqueous solutions or under very low levels of water potential. In these cases, we recommend gently scraping the surface across the entire embryo until all the seed coat and endosperm pieces come off. Damaged embryos (see Figures 8 and 9) should be removed from further analysis.
  e. Pipette the seed coat and endosperm debris out.
  f. Add 20–50 μL of the osmolyte solution or glycerin depending on the condition.
  g. Place a cover slip on top and proceed immediately to *Imaging.*
    ***Note:*** Rinse the tweezers and needle with water and wipe between each dissection to avoid the formation of osmolyte crystals upon evaporation.

2. Seed treatment.
  a. Seed imbibition:
    i. Submerge 2–3 seeds in 0.5–1 mL in one of the osmolyte solutions prepared in step 1. Ensure that the seeds sink to the bottom of the 1.5 mL Eppendorf tube by inverting it a few times.
    ii. Incubate for 15–30 min at room temperature (20°C–23°C).
  b. To examine the dry state of the embryo, submerge 2–3 seeds in 0.5–1 mL glycerin. Glycerin is a non-aqueous solution that, upon seed coat removal, will maintain the hydration state of the embryo as it was in the intact seed (Dorone et al., 2021). It also serves as an immersion medium for the embryo during imaging.

3. Seed dissection.
  a. Pipette one seed with approximately 5–10 μL of the solution in which it was submerged. Place on a cover slip or slide.
  b. Under a dissecting microscope (3× magnification), hold the seed between tweezer tips (Figure 1A).

### Physiologically relevant imbibition of the embryos

**Timing: variable (> 1 day)**

In this step, intact seeds are imbibed at physiologically relevant osmolyte concentrations. After this step, seeds are dissected in glycerin to preserve the hydration state of the embryos as it was prior to seed coat removal. As a result, the subcellular localization of the protein of interest can be assessed at the hydration state that is naturally achieved within intact seeds.

4. Fresh media preparation.
  a. Prepare standard growth medium:
    i. Prepare Murashige and Skoog (“MS”) medium using the following recipe:

**Table:** Murashige and Skoog medium

Reagent	Final concentration	Amount for 1L
Murashige & Skoog Basal Medium (PhytoTechnologies Laboratory)	0.5×	2.15 g
MES (Research Products International)	0.05%	0.5 g
Sucrose (Sigma-Aldrich)	1%	10 g
Agar (Difco)	0.8%	8 g

Before adding the agar, adjust the pH to 5.7 using potassium hydroxide.
    ii. Autoclave using a liquid cycle (20–30 min, 121°C).
      ***Note:*** Other growth media can be chosen depending on the study. For example, medium containing only water and agar, or MS medium without sucrose can be used.
  b. Prepare the osmolyte:
    i. Weigh the osmolyte of choice needed to generate a 5–10× stock medium. For NaCl, weigh 29.22 g (which will be used to make a 1M stock solution in step 4c).
    ii. Autoclave a bottle containing the weighed osmolyte using a dry cycle (20–30 min, 121°C).
  c. Prepare a stock medium by filling the autoclaved bottle from step 4b. with the media prepared in step 4a. to the required volume. In the case of NaCl, add MS medium to the 29.22 g from step 4b for a final volume of 500 mL to obtain a 1M NaCl solution.
    ***Note:*** While the osmolyte can be introduced directly in step 4a., we recommend separating these two steps as evaporation during autoclaving will concentrate the osmolyte and affect reproducibility.
  d. Use the stock medium to prepare diluted media at the desired concentrations. In the case of NaCl, the following range of concentrations can be prepared:

**Table:** Dilutions of MS medium supplemented with NaCl

Concentration	80 mM	120 mM	160 mM	200 mM	240 mM	280 mM
**1 M NaCl MS medium**	12 mL	18 mL	24 mL	30 mL	36 mL	42 mL
**MS medium**	138 mL	132 mL	126 mL	120 mL	124 mL	108 mL

    ***Note:*** We recommend using this approach of diluting a stock medium instead of preparing each medium individually from scratch. In our experience, even small variabilities during preparation affect the results, which makes the experiment less reproducible.
    ***Note:*** Here, 280 mM NaCl was chosen as the maximum concentration since Col-0 (WT) seeds will stop germinating at concentrations above 240 mM (Dorone et al., 2021). However, for other ecotypes or genotypes, this upper bound may differ.
  e. Plate the media in a sterile, laminar flow hood:
    i. To avoid evaporation after plating that may alter the final osmolyte concentration, cool the bottles to 50°C in a water bath set at 50°C.
    ii. Gently shake the bottle to mix its content, and pipette 40 mL into square petri dishes (120 × 120 wide × 15 mm high (VWR)).
    iii. Close the plates as soon as they dry (∼10 min) to avoid evaporation.

5. Seed sterilization. Work in a sterile, laminar flow hood
  a. Place 20–100 seeds in 1.5 mL Eppendorf tubes and add 1–1.5 mL of freshly prepared 70% ethanol (70:30 v/v ethanol:double-distilled water).
  b. Vortex for 5 min.
  c. Discard the solution and add 1 mL of 100% ethanol.
  d. Pipette the seeds onto pre-sterilized filter papers (e.g., Grade 410, VWR)
  e. Let the seeds dry on the filter papers for at least 20 min.
6. Seed imbibition.
  a. Place the seeds onto the agar plates prepared in step 4e by gently tapping them off the filter papers. Ensure that seeds are spaced out. Seeds that touch one another can be separated with a sterile toothpick.
  b. Seal the plates with micropore surgical tape (3M).
  c. Place the plates at the desired conditions. For example, seeds can be first stratified by placing the plates in the dark at 4°C for 3–5 days, and then placed in a growth cabinet at 22°C–23°C with 16 h light/8 h dark with 130 μmol m^−2^ s^−1^ PPFD and 50%–60% relative humidity.

**CRITICAL:** The purpose of this protocol is to assess protein localization before Phase III of water uptake (*i.e.,* during germination *sensu stricto*). It is important to ensure that seeds have not yet entered Phase III when imaging. For instance, under low osmolyte concentrations (e.g., < 80 mM NaCl), Col-0 seeds will typically start germinating within 24 hours when placed in otherwise optimal conditions (e.g., 22°C with illumination at 130 μmol m^−2^ s^−1^ PPFD). At that point, cells will have begun dramatic structural changes that will make comparisons with seeds placed under higher osmolyte concentrations less relevant. See Troubleshooting, problem 3 for an example of such changes.

7. To capture the hydration state of the seeds at a given time point, pick 2–3 seeds from the plates using a sterile toothpick or equivalent, and submerge in 0.5–1 mL glycerin. If the experiment involves a time series, work in sterile conditions and place the plate back in its experimental conditions until the next time point.
8. Dissect seeds as described in step 3 and proceed immediately to Imaging.

### Imaging

**Timing: 30–60 min**

In this step, we describe live embryo imaging using a Leica TCS SP8 laser scanning confocal microscope. While many of the steps described are specific to the Leica system, they can be easily adapted to use other confocal microscopes and software.

**Figure 2:**
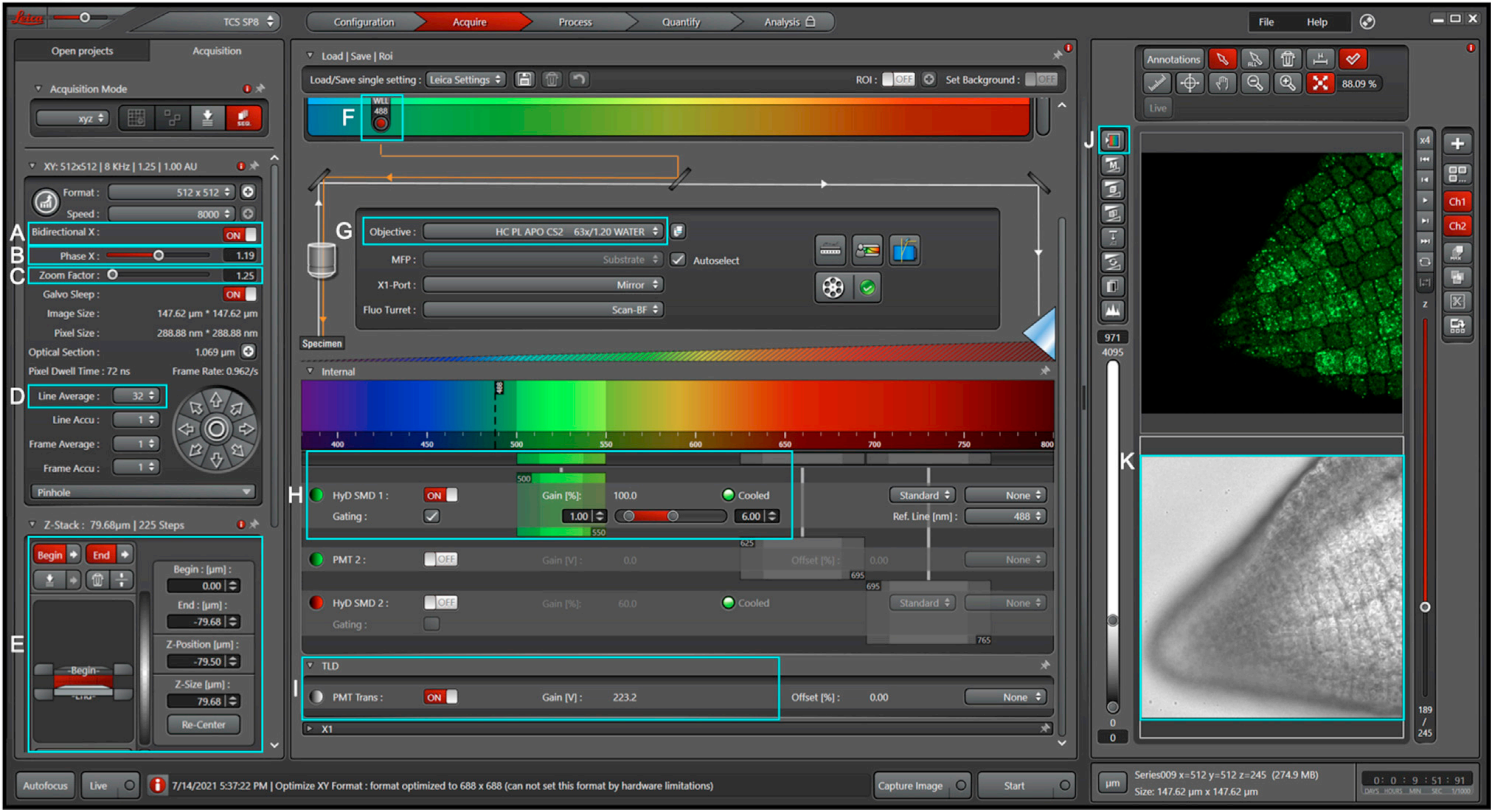
Initial imaging configurations on the Leica Application Suite X software
    ii. Set the excitation wavelength at 488 nm if using GFP (Figure 2F).
    iii. Select the HC PL APO CS2 63×/1.20 WATER objective if using samples mounted in aqueous solutions and 63/1.30 GLYCERIN if mounted in glycerin (Figure 2G).
    iv. Turn on one of the HyD SMD (Hybrid Detector Single Molecule Detection) detectors and set a detection interval of 50 nm starting at a wavelength at least 10 nm higher than the emission wavelength. In the case of GFP, set it at 500–550 nm (Figure 2H).
    v. Set the lifetime gate filter at 1.00–6.00 ns (Figure 2H).
      ***Note:*** Since most fluorescent proteins have fluorescence lifetimes within the 2.3–3.5 ns range (Mamontova et al., 2018), a lifetime gate filter of 1.50–6.00 ns can also be used.
    vi. Under TLD (Transmitted Light Detection) Turn on PMT Trans (transmission photomultiplier tube) (Figure 2I).

9. Software set up.
  a. Open the Leica Application Suite X software.
  b. Select resonant mode when prompted.
  c. “Configuration” settings:
    i. In the “Laser Config” tab, turn on the white light laser (WLL) and set at 70%.
    ii. In the “Hardware” tab, enable “Line Average during Live Acquisition” and set the “Bit Depth Resolution” at 12 bit.
    iii. In the “USB Panel” tab, select the “Fine” option for the “Phase” setting.
  d. “Acquire” settings:
    i. In the “Acquisition” tab, enable “Bidirectional X”, set the “Line Average” at 32, and set the “Zoom Factor” at 1.25 (Figures 2A, 2C, and 2D).
      ***Note:*** the line average can be set at lower or higher than 32 depending on the laser intensity, the fluorescence level of the protein of interest, and the desired resolution.

10. Image acquisition
  a. Load sample using the appropriate immersion media (water when using the HC PL APO CS2 63×/1.20 WATER objective and glycerin when using the 63/1.30 GLYCERIN objective).
    ***Note:*** We recommend starting with the wild-type ecotype (e.g., Col-0) and getting familiar with the protein storage vacuoles’ autofluorescence to avoid confusing these signals with those of the fluorescently tagged protein of interest. See Figure 3.
    **Figure 3:**
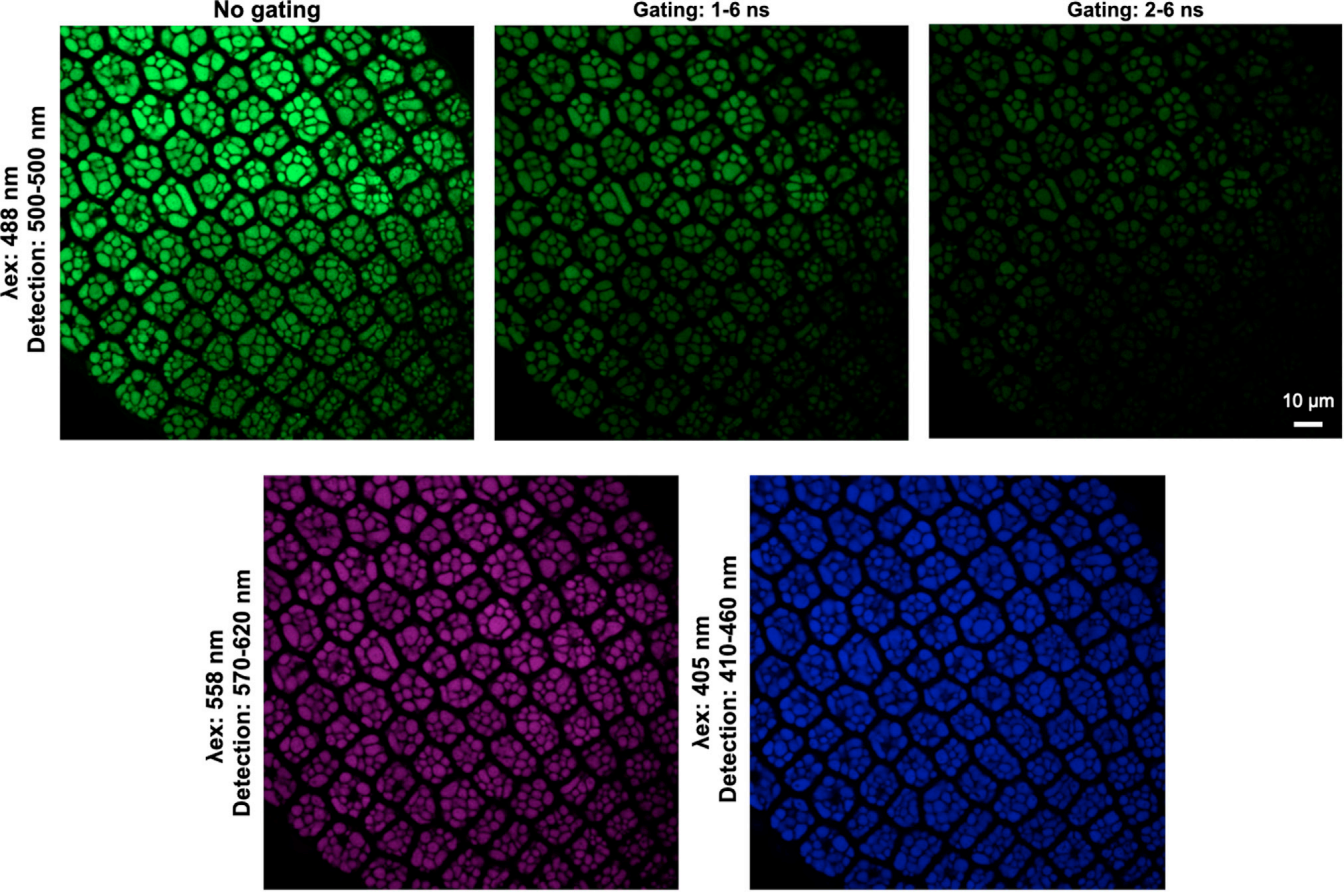
Protein storage vacuoles are autofluorescent A section of an embryonic radicle of Col-0 (WT) was imaged with different settings. Protein storage vacuoles are autofluorescent at imaging settings typically used for GFP imaging (top panels), RFP imaging (bottom left panel) as well as at the lower end of the visible spectrum (bottom right panel). Fluorescence lifetime gating of 1–6 ns (top middle panel) and of 2–6 ns (top right panel) can reduce autofluorescence. Single optical section images are shown.
  b. Find and focus on the sample. For example, in Figure 2K we centered on an embryonic radicle.
  c. Adjust the gain on the transmitted light channel (Figure 2I).
  d. Starting with a low laser intensity (e.g., 1% of the laser intensity), adjust intensity until the HyD SMD channel detects the fluorescence signal without reaching saturation which could damage the detector. One way of assessing saturation is to view the acquisition by its intensity values by clicking on the “Over-/underexposure” button (Figure 2J). The laser intensity can then be adjusted until only a minimal number of pixels appear blue.
    **CRITICAL:** When gating is turned on, the detector is exposed to a higher intensity than it appears on the software. To avoid damaging the detector, gating can be turned off while setting up the laser intensity.
  e. Adjust the phase so that the image appears clear (Figure 2B).
  f. Set the Z-Stack (Figure 2E).
  g. If needed, adjust the Line Average number (Figure 2D) so that the signal appears less grainy.
  h. Start image acquisition.

## Expected outcomes

This protocol aims to study the subcellular localization of a fluorescently tagged protein of interest under varying levels of water potential in *Arabidopsis thaliana* embryos.

In Figure 5, we give the example of how FLOE1’s localization is affected by different NaCl concentrations using the two methods described in this protocol. In Figure 5A, we show the results of the first approach that directly exposes naked embryos to varying levels of water potential (see the direct imbibition of embryos section*)*. FLOE1 forms granules across embryo radicles when exposed to water (Figure 5A, left panel). However, when salt is added at a concentration of 1 M, granules are less prominent and fewer cells exhibit them (Figure 5A, middle panel). At 2 M, granules can no longer be observed (Figure 5A, right panel). In Figure 5C, we employed the second approach (see the physiologically relevant imbibition of embryos section*)*, in which intact seeds (i.e., having their seed coats) were first imbibed on media supplemented with different osmolyte concentrations, after which they were dissected in glycerin to prevent further hydration and “capture” the hydration state of the embryos prior to seed coat and endosperm removal. Since the seed coat—which acts as a physical barrier—is present, FLOE1 granules are barely visible at concentrations of salt higher than 280 mM even after a total of six days (five days of stratification, one day of incubation), compared to 80 mM where they are more prominent. These differences can be quantified using the fluorescence heterogeneity score of each cell across three independent embryos for each condition. As shown in Figures 5B and 5D, the less cells exhibit FLOE1 granules the lower their heterogeneity scores.

## Quantification and statistical analysis

### FLOE1 case example

In this step, we use embryos expressing FLOE1p::FLOE1-GFP (Dorone et al., 2021) as an example of how to analyze cell-to-cell variations in protein localization. FLOE1 forms cytoplasmic granules whose intensity and number vary in response to water potential (Dorone et al., 2021). We anticipate that similar normalization methods could be used to study the partitioning of other proteins of interest into different compartments (e.g., nucleocytoplasmic partitioning).

1. Quantification
  a. Open the Quantification tab in the Leica Application Suite X software and open the saved Z-stack.
  b. Select the Channel used to acquire the fluorescence signal.
  c. Generate a maximum projection image by clicking on the MAX icon (Figure 4A).

**Figure 4:**
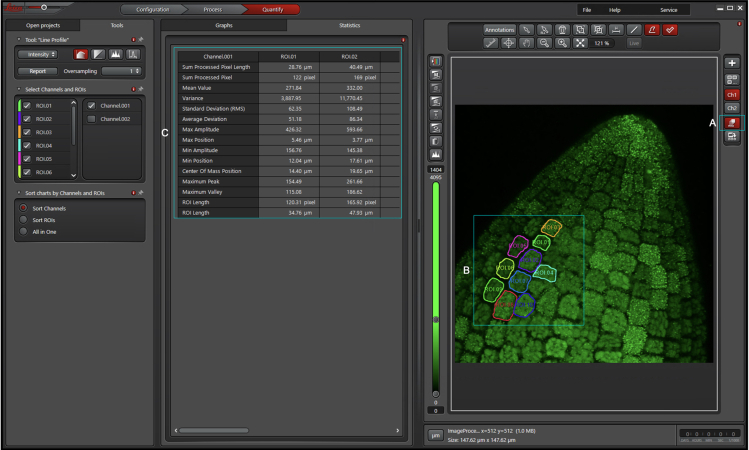
Image analysis using the Leica application suite X software
  d. Draw ROIs (Regions Of Interest) around each cell (Figure 4B). This will automatically generate data in the Statistics tab (Figure 4C).
  e. Export and save the Statistics table (Figure 4C).
2. Analysis
  a. Open the saved table in Excel.
  b. Divide the Standard Deviation (RMS) value by the Mean Value to obtain heterogeneity scores for each embryonic cell. These scores measure the normalized variability of fluorescence within each cell and, depending on the case, can serve as a proxy for assessing partitioning between different organelles. For example, in Dorone et al. (2021), we used this score as a proxy for the number of granules and their intensity (Dorone et al., 2021).
  c. Datasets for at least three embryos can then be used for comparison across different conditions (see Expected Outcomes). For example, these values can be visualized and analyzed on GraphPad Prism as shown in the examples provided in Figures 5B and 5D.

**Figure 5:**
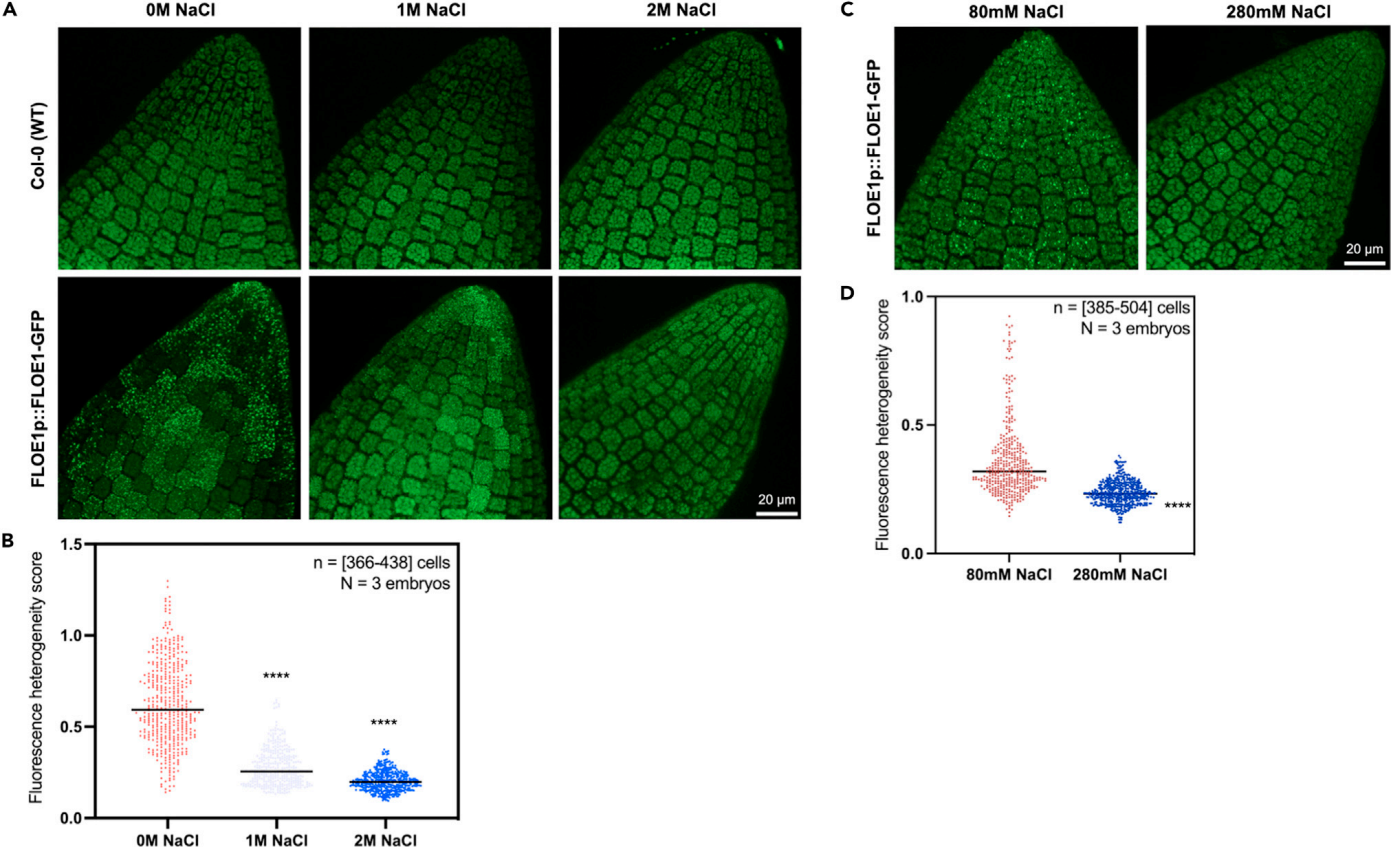
Imaging and quantification examples in embryos expressing FLOE1p::FLOE1-GFP (A) Col-0 embryos (top panels) and embryos expressing FLOE1::FLOE1-GFP (bottom panels) were imbibed and dissected as described in the direct imbibition of embryos section in water supplemented with NaCl at the following concentrations: 0 M (left panel), 1 M (middle panel) and 2 M (right panel). FLOE1 forms granules in a hydration-dependent manner. The vast majority of cells exhibits granules at 0 M NaCl, less so at 1 M NaCl, and no visible granules at 2 M NaCl. Images are maximum projections of embryo radicles. (B) Quantification results for (A) (see Quantification and statistical analysis*: FLOE1 case example*) for three embryos at each condition are plotted in GraphPad Prism (v. 8.4.1). Each dot represents the fluorescence heterogeneity score of an individual radicle cell. One-way ANOVA Kruskal Wallis. ∗∗∗∗ p-value < 0.0001. (C) Embryos expressing FLOE1::FLOE1-GFP were stratified for 5d (4°C, dark) and incubated for 24h (22°C, 130 μmol.m^−2^ s^−1^ PPFD) on MS medium supplemented with NaCl at 80 mM (left panel) or 280 mM (right panel). They were then dissected in glycerin as described in the physiologically relevant imbibition of embryos section. Unlike in (A), imbibition occurs through the seed coat, which alters the environmental water potential required for FLOE1 granules to appear. Images are maximum projections of embryo radicles. (D) Quantification results for (C) (see Quantification and statistical analysis*: FLOE1 case example*) for three embryos at each condition are plotted in GraphPad Prism (v. 8.4.1). Each dot represents the fluorescence heterogeneity score of an individual radicle cell. Mann-Whitney. ∗∗∗∗ p-value < 0.0001.

## Limitations

Autofluorescence of the protein storage vacuoles is a major obstacle when a protein is expressed below a low threshold. We recommend always comparing the images to the background ecotype (e.g., Col-0), and getting familiar with what constitutes autofluorescence versus real signals emitted by the fluorescent tag.

Additionally, as this procedure is invasive, the seeds cannot be reused for subsequent germination experiments. Therefore, it complicates experiments that aim to understand whether specific subcellular localization patterns are directly linked to the germination phenotype of a given seed.

## Troubleshooting

### Problem 1

No fluorescence signal can be detected other than the autofluorescent protein storage vacuoles.

### Potential solution

First, ensure that the protein is expressed in the seeds using a Western Blot or ELISA test (see (Dorone et al., 2021) for protein extraction and GFP ELISA using seeds). In our experience, certain promoters such as the 35S Cauliflower Mosaic Virus (CaMV) promoter may have sporadic expression levels in the embryo (see problem 2), with some transgenic lines not expressing the transgene at all (step 1).

Second, if the protein is indeed expressed, the signal may be drowned out by autofluorescence. In this case, we recommend trying different promoters that are highly expressed in the seed, but avoiding certain overexpression promoters like 35S CaMV (see the comment above as well as problem 2).

### Problem 2

Protein expression is patchy in the embryos.

### Potential solution

**Figure 6:**
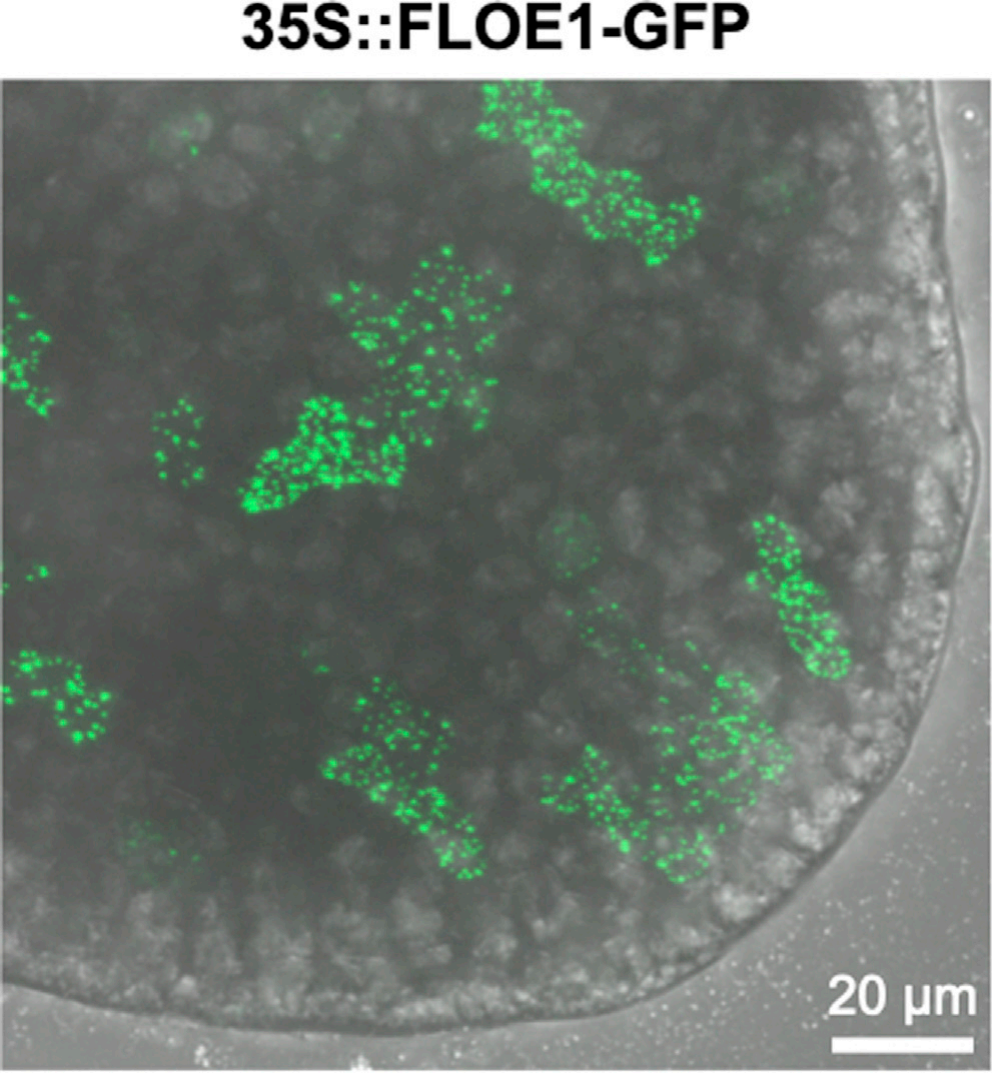
A line carrying a 35S::FLOE1-GFP transgene exhibits a patchy expression in *Arabidopsis* embryos An embryo carrying a 35S::FLOE1-GFP construct was imbibed and dissected in water as described in the direct imbibition of embryos section. Maximum projection image of a cotyledon (embryonic leaf). The GFP channel was merged with that of the transmitted light detector.

When using the 35S CaMV promoter, expression of certain proteins may be patchy in the embryos (Figure 6). We recommend using the native promoter or trying different promoters (e.g., known seed-specific promoters).

### Problem 3

The protein storage vacuoles seem abnormal or not well-defined.

### Potential solution

**Figure 7:**
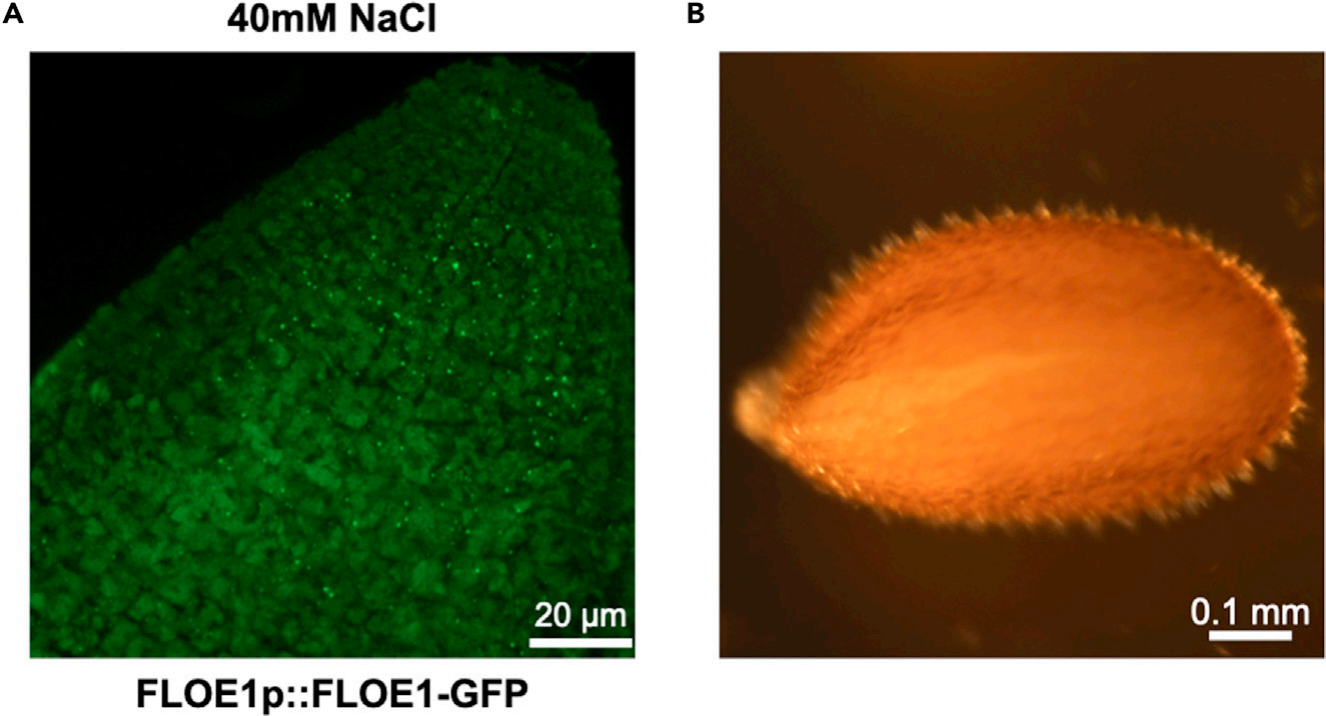
Protein storage vacuoles have a different appearance in embryos that have initiated germination (A) After 5 days of stratification (4°C, dark) on MS medium supplemented with 40 mM NaCl, seeds expressing FLOE1p::FLOE1-GFP were transferred to continuous light (130 μmol m^−2^ s^−1^ PPFD) for 24 h at 22°C. Embryos were then dissected out in glycerin as described in the physiologically-relevant imbibition of the embryos section. Image is a maximum projection of an embryo radicle. (B) Example of a seed crack that indicates that the radicle is about to emerge.

This is likely due to the embryos having entered Phase III of germination (step 6c). In the example shown in Figure 7A, seeds expressing FLOE1p::FLOE1-GFP were first sowed on 40 mM NaCl media as described in the physiologically-relevant imbibition of the embryos section. They were first stratified for 5 days at 4°C in the dark and then placed in light conditions for 24 h at 22°C with 130 μmol.m^−2^.s^−1^ PPFD. While seeds exposed to concentrations higher than 80 mM have not yet germinated at that point and do not appear to have reached Phase III yet, those at 40 mM are further along the process, as evidenced by seed cracks in the seed population (Figure 7B).

### Problem 4

Some cells appear dark.

### Potential solution

**Figure 8:**
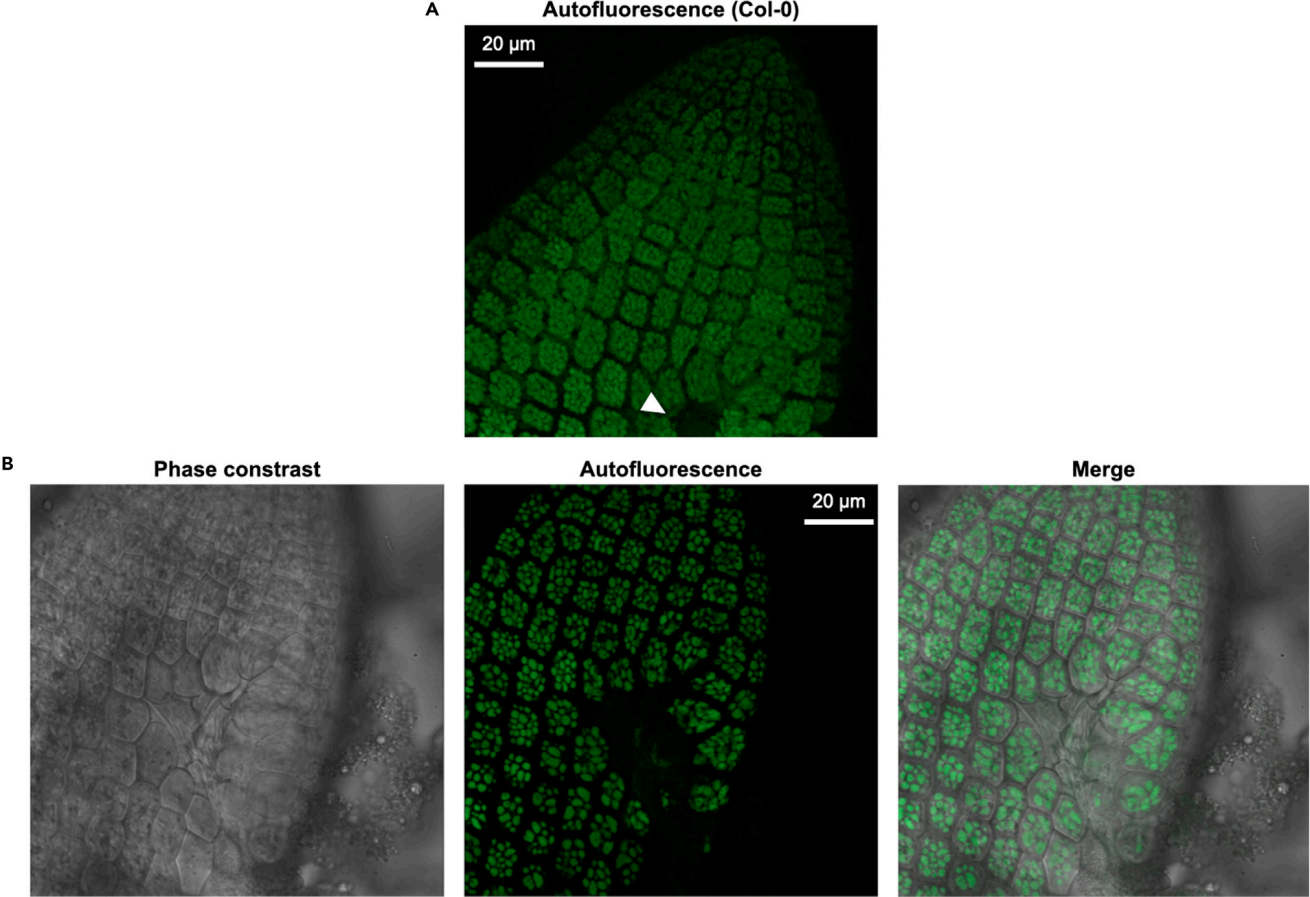
Example of a damaged embryo (A) A maximum projection image capturing the autofluorescence of a Col-0 (WT) embryonic radicle exhibits a “dark cell” (indicated by the arrowhead). (B) A single optical section of the dark region shown in (A) reveals tissue damage.

This often occurs upon accidental scratching or poking of the embryo during dissection (see Figure 8) (step 3c). To avoid this, we recommend making the incision in the seed coat along a tissue that will not be imaged (e.g., incision along the cotyledons if the radicle is to be imaged). Additionally, tungsten needles of a higher diameter can be used. While dissection will require more patience, it will also reduce the risk of damage.

***Note:*** we recommend discarding damaged samples from further analysis.

### Problem 5

**Figure 9:**
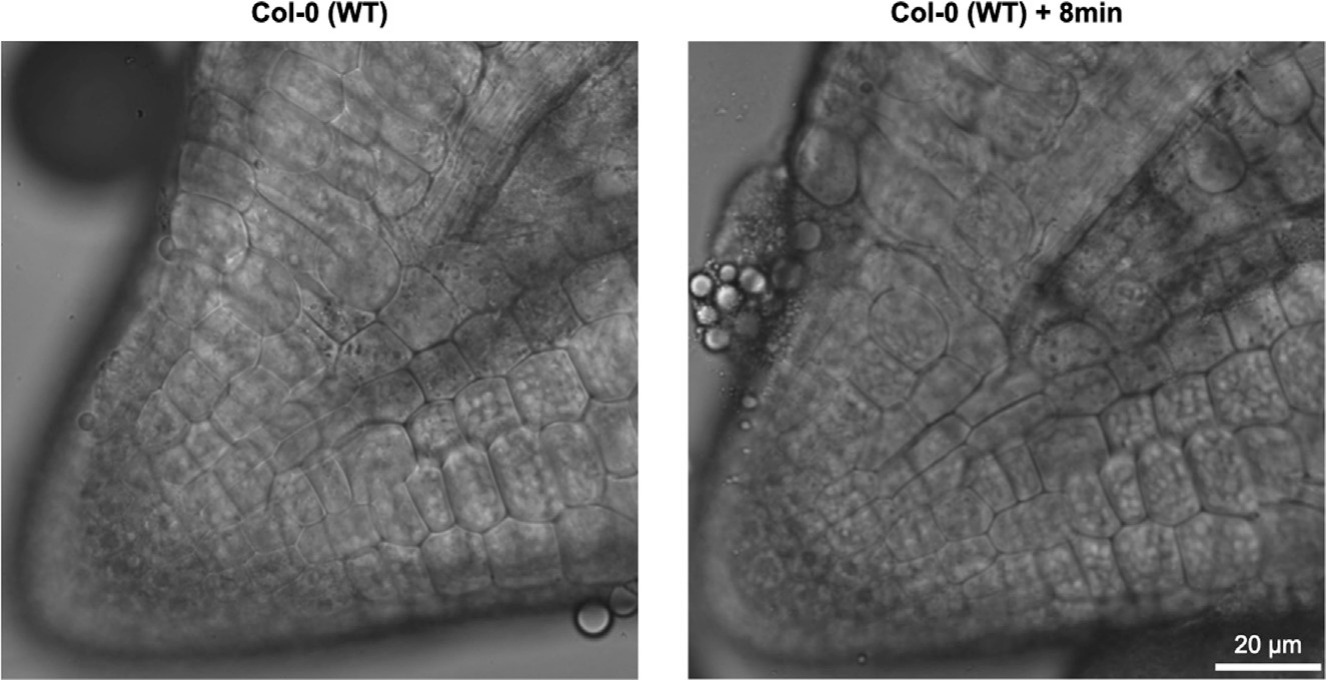
Example of an embryo that splits A Col-0 (WT) embryonic radicle imbibed in water splits longitudinally along the middle of the radicle. The same embryo (left) was imaged 8 min later (right) to show the progression of the damage. Single optical sections are shown.

Embryos split during imaging (Figure 9).

### Potential solution

In our experience, this happens when even minimal pressure is applied on the cover slip (step 3g). To avoid this, we recommend delicately placing the cover slip on top of the sample right before mounting it on the microscope instead of preparing the slides in advance and carrying them to the instrument.

## Resource availability

### Lead contact

Further information and requests for resources and reagents should be directed to and will be fulfilled by the lead contact, Seung Rhee (srhee@carnegiescience.edu).

### Materials availability

Plant lines used in this study will be made available on request from the lead contact upon completion of a Materials Transfer Agreement.

## Data Availability

This study did not generate new datasets or codes.
